# Have We a Homœpath among Us?

**Published:** 1853-07

**Authors:** 


From the New-Hampshire Journal of Medicine.

*Have we a Homœopath among us?*

(A SHOT in the right direction. May it provoke a discharge of the little pills?)—EDS. N. W. M. & S. JOUR.

There is a fair prospect that this question must be asked by the members of the National Medical Association at their meeting next month; for, strange as it may appear, this body of physicians are not secure from the presence of homœopathists or any other one-idea practitioners. To explain we quote the following paragraph, headed: "Legitimate Medicine and the Massachusetts Medical Society," from the Boston Medical Journal:

"Report says that a paper is about receiving the names of petitioners to the Mass. Medical Society, requesting the adoption of measures by which the homœopathic members of the Society shall be excluded from the same; and if such measures are not adopted, then it is stated the memorialists will themselves sever their connection with the Society. Perhaps rumor may have exaggerated the determination of the dissatisfied; but that something is contemplated of a decisive character, in relation to the homœopathists in our State Society is generally credited. Should a strong demonstration be made to thrust them out, it is impossible to divine the condition in which all parties will find themselves when the smoke of party feeling clears up. Several estimable, highly-educated young physicians, who have complied with every demand of the University, and studied under the best masters of medicine, have abandoned every precept and principle recognized in the schools, and turned homœopathic practitioners. What is to be their destiny? Are they empirics, irregular practitioners in every sense, and therefore justly denounced by those with whom they formerly associated? This, and similar questions, will naturally arise, and must be met without equivocation."

We have been aware of this condition of things in the Massachusetts Medical Society for some time, but as no statement of it has before met our eye, we have not felt at liberty to do more than allude to it. We have now the announcement of this anomalous state of affairs, under the authority of the venerable editor of the periodical from which this extract is made. Let us examine it, and consider how this affects other similar bodies.

It is evident, then, that there are in the Massachusetts State Medical Society certain men who are homœopathists; and, for all that appears to the contrary, they are in good standing. It also seems that there is a report "that a paper is about receiving the names of practitioners, requesting the adoption of measures by which the homœopathic members of the Society shall be excluded;" and report farther says, that if a measure is not adopted, then the memorialists themselves will sever their own connection with the Society. The writer is not sure that this determination is not exaggerated, but "it is generally credited" that something *of a decisive character* is contemplated. We rejoice that there is even a *rumour* that any members of that Society are unwilling longer to associate themselves in a medical society with those who deny the truth of the principles of medical science;—and do not at all understand how they have endured it so long. They are members of the same Society, but do they *consult* with them? Do they recognize them as physicians *in* the Society, and refuse to do so *out* of it? But, "should a strong demonstration be made to thrust them out, it is impossible to divine the condition in which all parties will find themselves, when the smoke of party feeling clears up;" that is to say, it is impossible to tell who will be successful in this conflict, the petitioners or the homœopaths. Is it not so? But are the homœopaths so numerous in that body that they can carry a majority? Or are those who would not tamper in any way with this form of quackery so few that they cannot expect to be successful? Still this body sends its delegates to the American Medical Association, one for every ten members. How many of these delegates at the next session will represent homœopathists? Or how many of these delegates will *be* homœopathists? If none, we shall be glad; but what security is there that there are, or will be none? Is it right, is it honorable for a Society which retains amongst its members those who are avowed homœopathists, or any other sectaries in medicine, to send delegates to meet those from other similar associations, which at once reject any and every man who thus proves a renegade to all true science? It is impossible, then, to deny that a homœopathist may claim a seat in the National Medical Association as justly and as securely as any other delegate from the Massachusetts State Medical Society. If the members of the Association are true to themselves, this danger need not again occur; but if they falter, their seats may be occupied by all kinds of empirics, and their Society lose all its influence and authority.

Our neighbor, from whom we copy, seems to be a little puzzled as to what to do with "several estimable, highly-educated young physicians, with every requisite of the (Cambridge?) University," etc. Are they any the less "empirics, irregular practitioners in every sense, and therefore justly denounced," because they have had unusual advantages? We apprehend not, but are the more to be blamed that they have thus turned away, after having been made familiar with the instructions of science. True, such questions as these are to be met without equivocation, and we feel proud that we at least are not compelled to blow hot and to blow cold to retain subscribers, or to fill the pocket of our publisher, or in any other way to dally with this or any other form of charlatanism.

